# *Ferrimicrobium acidiphilum* Exchanges Electrons With a Platinum Electrode *via* a Cytochrome With Reduced Absorbance Maxima at 448 and 605 nm

**DOI:** 10.3389/fmicb.2021.705187

**Published:** 2021-07-26

**Authors:** Robert C. Blake, Amit Nautiyal, Kayla A. Smith, Noelle N. Walton, Brealand Pendleton, Zhe Wang

**Affiliations:** ^1^Division of Basic Pharmaceutical Sciences, College of Pharmacy, Xavier University of Louisiana, New Orleans, LA, United States; ^2^Department of Chemistry, Xavier University of Louisiana, New Orleans, LA, United States; ^3^Department of Chemistry, Oakland University, Rochester, NY, United States

**Keywords:** *Ferrimicrobium acidiphilum*, spectroelectrochemistry, cyclic voltammetry, chronocoulometry, electron transfer, integrating cavity absorption meter, standard reduction potential

## Abstract

*Ferrimicrobium acidiphilum* is a Gram-positive member of the *Actinobacteria* phylum that can respire aerobically or anaerobically with soluble Fe(II) or Fe(III), respectively, in sulfuric acid at pH 1.5. Cyclic voltammetry measurements using intact *F. acidiphilum* at pH 1.5 produced fully reversible voltammograms that were highly reproducible. The maximum current observed with the anodic peak was considerably less than was the maximum current observed with the cathodic peak. This difference was attributed to the competition between the platinum electrode and the soluble oxygen for the available electrons that were introduced by the cathodic wave into this facultative aerobic organism. The standard reduction potential of the intact organism was determined to be 786 mV vs. the standard hydrogen electrode, slightly more positive than that of 735 mV that was determined for soluble iron at pH 1.5 using the same apparatus. Chronocoulometry measurements conducted at different cell densities revealed that the intact organism remained in close proximity to the working electrode during the measurement, whereas soluble ionic iron did not. When the cyclic voltammetry of intact *F. acidiphilum* was monitored using an integrating cavity absorption meter, the only small changes in absorbance that were detected were consistent with the participation of a cellular cytochrome with reduced absorbance peaks at 448 and 605 nm. The cytochrome that participated in the exchange of electrons between the intact organism and extracellular solid electrodes like platinum was the same cytochrome whose oxidation was previously shown to be rate-limiting when the organism respired aerobically on extracellular soluble iron.

## Introduction

The capacity to respire aerobically on soluble ferrous ions at pH values less than 3.0 is currently thought to be expressed in 42 species distributed among 19 genera in 6 phyla (Johnson et al., 2012). These 6 phyla are equally distributed among the Gram-negative eubacteria, the Gram-positive eubacteria, and the archaea. A majority of these microorganisms also exhibit the capacity to respire anaerobically on soluble ferric ions at acidic pH values using molecular hydrogen (Drobner et al., 1990; Ohmura et al., 2002b; Hedrich and Johnson, 2013), reduced sulfur compounds (Brock and Gustafson, 1976; Pronk et al., 1991; Osorio et al., 2013), or selected organic molecules as electron donors (Weber et al., 2006; Johnson and Hallberg, 2009). *Ferrimicrobium acidiphilum* is a member of the Gram-positive *Actinobacteria* that catalyzes both the dissimilatory oxidation and reduction of soluble iron in the presence of suitable organic carbon (Johnson et al., 2009). *In situ* absorbance measurements were conducted on intact *F. acidiphilum* using an integrating cavity absorption meter that permitted accurate absorbance measurements in turbid suspensions of the cells in the presence of soluble ferrous ions (Blake et al., 2020). A single cytochrome with reduced absorbance peaks at 448 and 605 nm was the only cellular chromophore that exhibited readily detected absorbance changes as the cells respired aerobically on reduced iron. The reduced cytochrome 605 possessed the functional properties that were consistent with the hypothesis that oxidation of the cytochrome was the rate-limiting step as *F. acidiphilum* respired aerobically on iron.

Over 50 years ago, investigators attached both leads from a 6-V battery charger to electrodes immersed in the same growth medium and demonstrated a 6-fold increase in the batch yield of *Ferrobacillus sulfooxidans* (now *Acidithiobacillus ferrooxidans*) cultured on soluble iron (Kinsel and Umbreit, 1964). Thirty years later, our laboratory described an electrochemical apparatus for culturing chemolithotrophic bacteria that respire aerobically on ferrous ions in which greatly enhanced yields of the bacteria were achieved by the *in situ* electrochemical reduction of soluble iron in the growth medium (Blake et al., 1994). Eight years later, investigators described a similar apparatus for culturing chemolithotrophic bacteria that respire anaerobically on ferric ions in which greatly enhanced yields of the bacteria were achieved by the *in situ* electrochemical oxidation of soluble iron in the growth medium (Ohmura et al., 2002a). If acidophilic chemolithotrophs that exchange electrons with soluble iron can be cultured electrochemically by keeping the soluble iron in the appropriate oxidation state, then it is evident that it should be possible to conduct electrochemical measurements on such organisms in the presence of soluble iron if one has the means to purposefully manipulate and monitor the oxidation state of the soluble iron. Further, one can hypothesize that it is possible to conduct spectroelectrochemical measurements on the same chemolithotrophs if one also has the means to conduct accurate absorbance measurements in turbid suspensions of the intact microorganisms. The latter hypothesis was tested herein using *F. acidiphilum* as the test organism.

The experiments described below had 2 principal objectives: (i) to measure the reduction potential(s) of intact *F. acidiphilum* under physiological conditions for the organism; and (ii) to identify any colored biomolecules that changed their redox status when the intact cells were undergoing oxidation or reduction. These experiments were conducted with the initial expectation that electrochemical measurements on intact *F. acidiphilum* would require soluble iron to mediate the transfer of electrons between the bacterium and the surface of an electrode in any electrical circuit imposed by a potentiostat. Electrochemical measurements on soluble iron yielded useful information, including the actual surface area of the platinum mesh working electrode. It became immediately apparent, however, that the intact bacterium reproducibly exchanged electrons directly with the surface of the electrode in the absence of soluble iron or any other extracellular or exogenous electron transfer mediators. Cyclic voltammetry and chronocoulometry measurements were consistent with the hypothesis that the cells rapidly coated the surface of the solid electrode and exhibited a single reduction potential whose value was between those of soluble iron and molecular oxygen. A customized combination of absorbance cuvette and electrochemical cell was employed to monitor absorbance changes that occurred with colored electron transfer proteins in the intact cells during cyclic voltammetry manipulations. The only reduced cellular protein that was visible during the cathodic phase of the cyclic voltammetry measurements was a cytochrome with reduced peaks at 448 and 605 nm, which appeared to be the same cytochrome as that identified previously when the cells respired aerobically on soluble iron (Blake et al., 2020).

## Materials and Methods

### Cell Culture and Quantification of Bacteria

*Ferrimicrobium acidiphilum* DSMZ 19497^*T*^ was cultured aerobically with agitation (120 revolutions per minute) on the mixotrophic medium (20 mM ferrous sulfate plus 0.2% (w/v) yeast extract, pH 1.8) at 30°C, as described previously (Blake et al., 2020). Cells grown to late stationary phase with regard to ferric ion production were then cycled at least three times, where the culture was permitted to sit without agitation overnight while a portion of the ferric iron in solution was reduced back to the ferrous state. Agitation of the culture resumed in the morning, and the subsequent reoxidation of the reduced iron that had accumulated overnight completed one “cycle.” Cells treated in this manner were harvested by centrifugation, washed five times with 0.01°N H_2_SO_4_, and resuspended in sufficient sulfuric acid, pH 1.5, to achieve a stock suspension in excess of 10^10^ cells/ml. The stock suspensions were stored at 4°C. Electrochemical and/or spectroscopic experiments were conducted on aliquots of the cells within 1 week following harvesting.

Absolute numbers of *F. acidiphilum* cells were determined by electrical impedance measurements on a Multisizer 4 particle counter (Beckman Coulter, Inc., Bria, CA, United States) fitted with a 30-μM aperture (Blake et al., 2020). The instrument was programmed to siphon 50 μl of sample that contained Isoton II as the electrolyte. The current applied across the aperture was 600 μA. Voltage spikes attendant with impedance changes as microorganisms passed through the aperture were monitored with an instrument gain of four.

### Electrochemical Measurements

Cyclic voltammetry (CV) and chronocoulometry (CC) measurements were conducted using a Model 1100A Electrochemical Analyzer/Potentiostat from CH Instruments, Inc. (Austin, TX, United States). A single compartment electrochemical cell with a 3-electrode system was used where a platinum wire was the counter electrode, a platinum mesh was the working electrode, and silver/silver chloride was the reference electrode (4 mm outer diameter, #932-00018), all from Gamry Instruments, Inc. (Warminster, PA, United States). The electrodes were inserted into a UV-Vis Spectroelectrochemical Cell Kit (Gamry) through a cuvette lid that was custom-designed to accommodate all three electrodes within a standard, rectangular 3.5 ml quartz fluorescence cuvette.

Cyclic voltammetry measurements were routinely conducted in 2 ml of analyte solution/suspension that was placed in the quartz cuvette. The silver/silver chloride reference electrode was calibrated for CV measurements using a 50 mM solution of equimolar ferri- and ferrocyanide in 0.1 M KCl within the potential range of −0.6 to 1.2 V (∼−0.2 to 1.6 V vs. the SHE) at a scan rate of 256 mV/s. All CV measurements in either acidic soluble iron or suspensions of intact cells were conducted in the potential range of −0.2 to 1.6 V vs. the SHE. Unless noted otherwise, each individual CV measurement consisted of a minimum of 5 complete cycles. The first cycle from each measurement was discarded due to stabilization of electrodes.

Double potential step CC measurements were also routinely conducted in 2 ml of analyte solution/suspension. The first pulse was a cathodic step from 1.6 to −0.2 V vs. the SHE; the second pulse was an anodic step from −0.2 to 1.6 V vs. the SHE. Parameters for CC measurements in either acidic soluble iron or suspensions of intact cells were as follows: pulse width, 1.0 s; sample interval, 5 ms; sensitivity, 0.04 A/V; and quiet time, 2 s. Five successive repetitions were performed for each individual double potential CC measurement.

### Absorbance Measurements With Cell Suspensions

Absorbance measurements on intact *F. acidiphilum* in suspension were conducted in an OLIS CLARiTY 1000A spectrophotometer (On Line Instrument Systems, Inc., Bogart, GA, United States) that employed a novel integrating cavity absorption meter (Blake and Griff, 2012; Li et al., 2015; Blake et al., 2020). In a typical measurement, identical 8-ml solutions that contained sulfuric acid, pH 1.5, were added to both the sample and the reference observation cavities of the spectrophotometer. When the focus of the observation was the production of ferric ions, both the sample and reference cavities initially contained the same concentration of ferrous sulfate. After a stable baseline was recorded from 290 to 437 nm, a small sample was withdrawn from the sample cavity and replaced with an equal volume of suspended *F. acidiphilum* to initiate subsequent reactions and absorbance changes within the cavity. When the focus of the observation was the effect(s) of ferrous ions on colored redox-active components within the intact microorganism, both observation cavities initially contained equal suspensions of intact *F. acidiphilum*. After a stable baseline was recorded from 417 to 631 nm, a small sample was withdrawn from the sample cavity and replaced with an equal volume of concentrated ferrous sulfate to initiate subsequent reactions and absorbance changes within the cavity.

Spectroelectrochemical measurements were conducted in the CLARiTY using a custom observation chamber that was designed to accommodate a standard, rectangular quartz cuvette. A rectangular micro quartz cuvette was employed that possessed dual paths of 2.0 and 10 mm (#52-Q-2, Starna Cells, Inc., Atascadero, CA, United States). A white cuvette lid was customized by OLIS to accommodate the 3-electrode system described above. Concomitant CV and absorbance measurements were conducted on 0.8 ml of a suspension of intact cells in sulfuric acid, pH 1.5, at 30°C. After recording a stable baseline from 417 to 631 nm, absorbance measurements were conducted at a rate of 6.2/s during the 6-s intervals of the cathodic portions of 10 repetitive cycles of CV measurements from 0.8 to 0.2 V vs. the SHE.

Regardless of the type of absorbance experiment, raw absorbance spectra that were obtained in the CLARiTY were converted to equivalent absorbance values/cm using Fry’s method (Fry et al., 2010) with analysis software provided by OLIS, Inc.

## Results

### Oxidation/Reduction Capabilities of Cells

*Ferroplasma acidiphilum* was reported to both oxidize soluble iron under oxic conditions and reduce soluble iron in the presence of selected exogenous electron donors under anoxic solution conditions (Johnson et al., 2009). For that reason, *F. acidiphilum* was deemed to be a good candidate upon which to conduct electrochemical measurements using soluble iron to mediate electron transfer between the organism and the surface of a solid platinum electrode. This laboratory reported that the oxidation of a cellular cytochrome with reduced absorbance peaks at 448 and 605 nm was rate-limiting when *F. acidiphilum* respired aerobically on ferrous ions (Blake et al., 2020). Nothing has been reported about the mechanisms and biomolecules that are involved in the reduction of soluble iron by the same organism. In particular, we don’t know whether different biomolecules are expressed for the oxidation and the reduction of soluble iron. If different biomolecules are expressed for the dissimilatory oxidation and reduction of iron, are both types of activities expressed constitutively or do they have to be induced? If different biomolecules must be induced, then what conditions are necessary to ensure that such inductions and/or expressions have occurred?

The spectra presented in Supplementary Figure 1 demonstrate that the cells that we harvested for these experiments could conduct both the oxidation and reduction of soluble iron. *Spectrum a* shows the absorbance spectrum associated with approximately 95% oxidation of 2.0 mM ferrous ions that occurred when *F. acidiphilum* was inoculated into an appropriate minimal mixotrophic growth medium and agitated for 3 days at 30°C. Ferric ion has an absorption coefficient of 890 M^–1^cm^–1^ at 340 nm in sulfuric acid, pH 1.5; ferrous ion has minimal absorbance at that wavelength in the same medium (Blake et al., 2020). The culture that yielded the sample for *spectrum a* in Supplementary Figure 1 was then permitted to incubate at the same temperature for an additional 16 h with no agitation. At that time, approximately 50% of the ferric ions was reduced back to the ferrous state (*spectrum b* in Supplementary Figure 1). The hypothesis was that the cell suspension became anoxic as aerobic respiration continued at a rate that outpaced the diffusion of new molecular oxygen molecules into the stationary culture. The likely source of electrons for that anoxic reduction of iron was hypothesized to come from organic sources of electrons within the 0.2% (w/v) yeast extract that was included in the minimal mixotrophic growth medium. When agitation resumed after the 16 h of rest, the soluble iron in the suspension was rapidly oxidized in just a few hours back to the level represented by *spectrum a* in Supplementary Figure 1. This cycle was repeated 3 times before the culture was harvested after the third period of iron reduction. Because the soluble iron was partially reduced at the time of harvesting, we were confident that the biomolecules necessary for iron reduction were expressed and active in the culture. Because any reduced iron in the culture was rapidly and reproducibly reoxidized if agitation was subsequently resumed, we were equally confident that the biomolecules necessary for iron oxidation were also expressed and active in the culture. Consequently, we were confident that cells cultured and harvested in this manner could perform both the oxidation and reduction of soluble iron.

### Calibration of Reference Electrode

**FIGURE 1:**
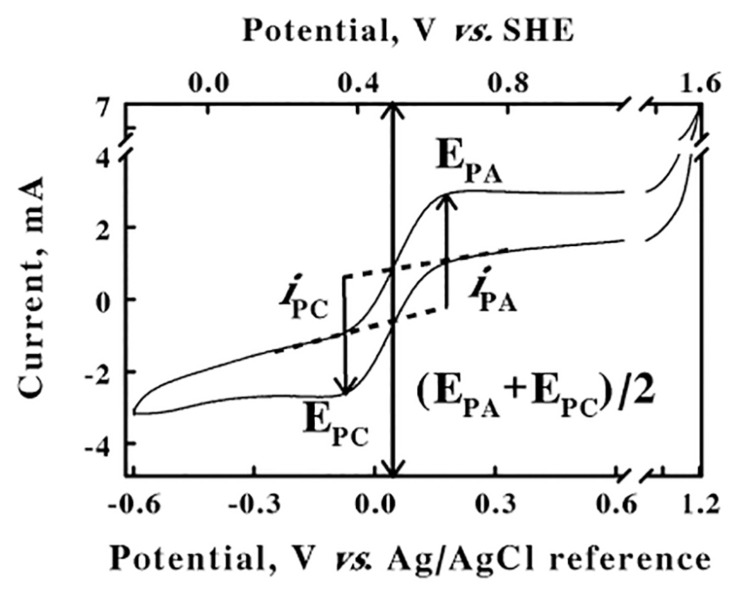
Calibration of the silver/silver chloride reference electrode. Cyclic voltammetry was conducted at a scan rate of 256 mV/s on a mixture of 25 mM each of ferri- and ferrocyanide in water. E_*PA*_, E_*PC*_, and (E_*PA*_ + E_*PC*_)/2 represent the potentials for the anodic and cathodic peaks and the ferri-/ferrocyanide couple, respectively; *i*_*PA*_ and *i*_*PC*_ represent the currents for the anodic and cathodic peaks, respectively. SHE represents the reduction potential for the standard hydrogen electrode.

**FIGURE 2:**
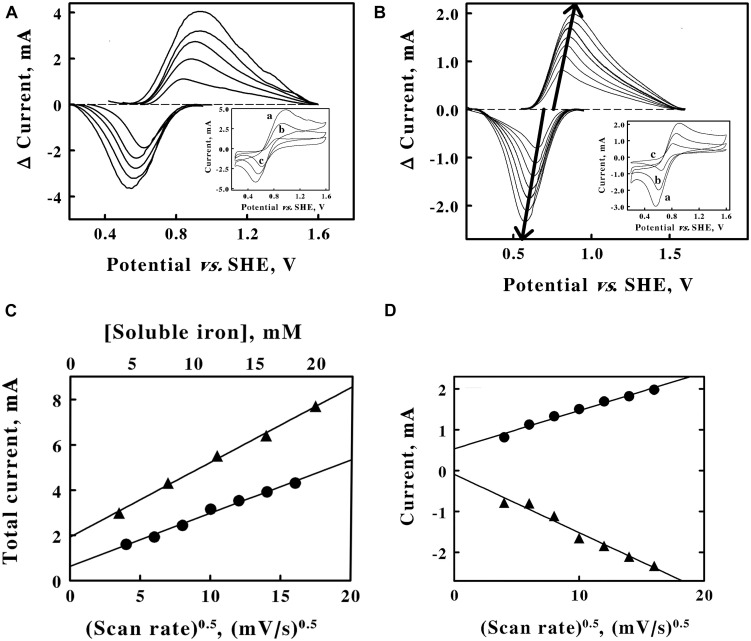
Cyclic voltammetry on soluble iron in sulfuric acid, pH 1.5. *A*, baseline-subtracted anodic (*positive*) and cathodic (*negative*) peak currents obtained with a scan rate of 256 mV/s at 5 concentrations of ferrous sulfate: 4, 8, 12, 16, and 20 mM, from lower to higher magnitude currents, respectively. *Inset*, representative cyclic voltammograms obtained with 20, 12, and 4 mM ferrous sulfate for *curves a*, *b*, and *c*, respectively. Each of the five cyclic voltammograms in **panel A** represents the mean of four highly reproducible individual measurements whose values varied by less than 1% among the four measurements. **(B)** Baseline-subtracted anodic (*positive*) and cathodic (*negative*) peak currents obtained with 8 mM ferrous sulfate at 7 scan rates: 16, 36, 64, 100, 144, 196, and 256 mV/s, from lower to higher magnitude currents, respectively. Each *bold arrow* passes through the peak of the current associated with each *curve*. *Inset*, representative cyclic voltammograms obtained at scan rates of 256, 100, and 16 mV/s for *curves a*, *b*, and *c*, respectively. Each of the seven cyclic voltammograms in **panel B** represents the mean of four highly reproducible individual measurements whose values varied by less than 1% among the four measurements. **(C)**, secondary plots of the total peak currents as a function of the soluble iron concentration (*triangles, upper abscissa*) and the square root of the scan rate (*circles, lower abscissa*) using data extracted from **panels A,B**, respectively. **(D)**, values for the anodic (*circles*) and cathodic (*triangles*) currents as a function of the square root of the scan rate. Each datum in **panels C,D** represents the mean of four determinations. In all cases, the standard deviation of each datum was within the area bound by the diameter of the datum in the figure.

The electrochemical potentials that were employed and/or measured in the cyclic voltammetry and chronocoulometry experiments performed below were all based on the stable potential provided by the silver/silver chloride reference electrode that was part of the 3-electrode system described in the Materials and Methods. This reference electrode was calibrated for the apparatus and experimental solution conditions imposed herein using the well-characterized ferricyanide/ferrocyanide couple (Murray and Rock, 1968) as a standard reference reaction. Figure 1 shows a cyclic voltammogram that was obtained from an equimolar mixture of ferri- and ferrocyanide (50 mM total concentration) in 0.1 M KCl, from an initial value of −0.6 V to a predetermined limit of 1.2 V. The cyclic voltammogram shown in Figure 1 represents an average of four highly reproducible individual measurements. The values of the anodic and cathodic currents at each potential varied by less than 1% among the four measurements. The *lower abscissa* represents the potential reported by the silver/silver chloride reference electrode. The anodic peak potential of 0.2 V, E_*PA*_, was the potential where the difference, *i*_*PA*_, between the actual current on the anodic (*upper*) sweep and the extrapolated baseline current was a maximum value. Likewise, the cathodic peak potential of −0.08 V, E_*PC*_, was the potential where the absolute value of the difference, *i*_*PC*_, between the actual current on the cathodic (*lower*) sweep and the respective extrapolated baseline current was also a maximum value. The reduction potential for the ferricyanide/ferrocyanide couple was calculated as (E_*PA*_ + E_*PC*_)/2, the mean of the anodic and cathodic peak potentials, of 0.06 V. The ferricyanide/ferrocyanide reduction potential in aqueous solution is generally acknowledged to be +436 mV vs. the standard hydrogen electrode (SHE)^1^ (O’Reilly, 1973). Consequently, the *upper abscissa* in Figure 2 represents the potentials vs. the SHE that were calculated by adding 0.376 V (0.436–0.06) to the potentials measured directly using the silver/silver chloride reference electrode in our electrochemical system. The potentials in all subsequent results in this paper are reported vs. that of the SHE.

### Cyclic Voltammetry of Soluble Iron

Cyclic voltammetry was conducted on soluble iron in sulfuric acid at pH 1.5 for two reasons: to verify that the calibrated reference electrode permitted one to accurately measure the reduction potential of other analytes; and to characterize the behavior of the ferric/ferrous couple in our electrochemical setup if and/or when we want to employ soluble iron to mediate electron transfer between a platinum electrode and intact chemolithotrophic microorganisms that normally exploit soluble iron as part of their energy metabolism.

The concentration of the analyte and the scan rate are two parameters that are routinely varied to gain insights into the electron transfer events in question (Kissinger and Heineman, 1983; Mabbott, 1983; Elgrishi et al., 2018). The *inset* of Figure 2A shows 3 representative cyclic voltammograms that were obtained at the same scan rate and 3 different concentrations of soluble iron. As the concentration of soluble iron increased over a 5-fold range, so too did the area bound by the *upper* anodic portion of the cycle and the respective *lower* cathodic portion of the cycle for each voltammogram. Each voltammogram had clearly defined anodic and cathodic peaks that differed significantly from their respective baselines. The *main panel* of Figure 2A shows the baseline-subtracted anodic and cathodic peaks that were extracted from their respective complete voltammograms. The extents of both the anodic and the cathodic currents at their respective peaks increased as the concentration of soluble iron increased.

The *inset* of Figure 2B shows 3 representative cyclic voltammograms obtained using the same concentration of soluble iron and 7 different sweep scan rates that varied over a 16-fold range. Once again, each voltammogram had clearly defined anodic and cathodic peaks that differed significantly from their respective baselines. The *main panel* of Figure 2B shows the baseline-subtracted anodic and cathodic peaks that were extracted from their respective voltammograms. The *bold arrows* emphasize that the extents of both the anodic and cathodic currents at their respective peaks increased as the scan rates increased. The *bold arrows* also pass through the peak values of the currents associated with each baseline-subtracted curve.

**FIGURE 3:**
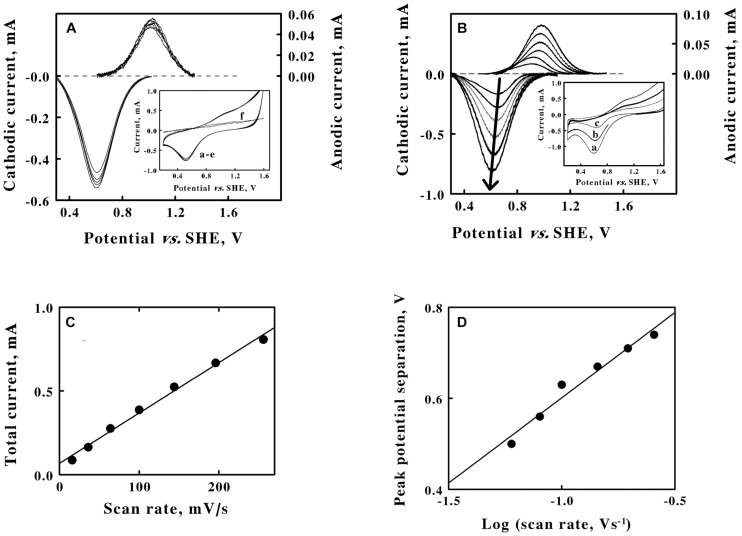
Cyclic voltammetry on suspensions of intact *F. acidiphilum* in sulfuric acid, pH 1.5. **(A)** baseline-subtracted anodic (*positive, right ordinate*) and cathodic (*negative, left ordinate*) peak currents obtained with a scan rate of 256 mV/s at 5 suspensions of cells: 0.48, 0.96, 1.44, 1.92, and 2.4 × 10^9^ cells/m. *Inset*, complete cyclic voltammograms for the same 5 suspensions in *curves a* through *e*; all 5 voltammograms are essentially superimposable. Each of the five cyclic voltammograms in **panel A** represents the mean of four highly reproducible individual measurements whose values varied by less than 1% among the four measurements. *Curve f* is the cyclic voltammogram that was obtained using growth media that remained when the intact cells were removed by a combination of centrifugation and filtration. **(B)** Baseline-subtracted anodic (*positive, right ordinate*) and cathodic (*negative, left ordinate*) peak currents obtained with 1.44 × 10^9^ cells/ml and 6 scan rates: 36, 64, 100, 144, 196, and 256 mV/s, from lower to higher magnitude currents, respectively. The *bold arrow* passes through the peaks of the currents associated with the *cathodic curves*. *Inset*, representative cyclic voltammograms obtained at scan rates of 256, 144, and 64 mV/s for *curves a*, *b*, and *c*, respectively. Each of the six cyclic voltammograms in **panel B** represents the mean of four highly reproducible individual measurements whose values varied by less than 1% among the four measurements. **(C)** Secondary plot of the total peak current as a function of the scan rate using data extracted from **panels A,**, respectively. **(D)** Secondary plot of the peak potential separation as a function of the logarithm of the scan rate using data extracted from **panel B**. Each datum in **panels C,D** represents the mean of four determinations. In all cases, the standard deviation of each datum was within the area bound by the diameter of the datum in the figure.

The reduction potential for the ferric/ferrous couple was calculated by averaging the means of the potentials that corresponded with each set of anodic and cathodic peaks associated with each of the 12 voltammograms shown in Figures 3A,B (Supplementary Table 1). The average value for the reduction potential of the ferric/ferrous couple in sulfuric acid at pH 1.5 was determined as 735 ± 20 mV vs. the SHE. The generally accepted value for the standard reduction potential of the ferric/ferrous couple in water is 770 mV (Ingledew and Cobley, 1980). That potential represents an extrapolated value in the absence of anions that might bind preferentially to soluble iron in either oxidation state. The sulfate anions (SO_4_^2–^, HSO_4_^1–^) that were present in our solutions at pH 1.5 bind more tightly to the ferric ion than they do to the ferrous ion (Blake and Shute, 1987). Consequently, one would expect the reduction potential for the ferric/ferrous couple to be slightly lower than 770 mV in sulfuric acid at pH 1.5, which is precisely what we observed.

Figure 2C shows secondary plots of the sum of the currents at their respective peaks (anodic plus cathodic) as a function of the soluble iron concentration (*triangles*) and the square root of the scan rate (*circles*). The two linear relationships are consistent with the predictions taken from the Randles-Sevcik equation (Elgrishi et al., 2018):

(1) $$i_{P}=0.4463\mathrm{nFAC}{\left(\mathrm{nFD}\upsilon /\mathrm{RT}\right)}^{0.5}$$

where *i*_*P*_ is the peak current, n is the number of electrons transferred per molecule (1 for the ferric/ferrous couple), F is Faraday’s constant, A is the surface area of the working electrode, C is the bulk concentration of the redox-active analyte, D is the diffusion coefficient of the analyte, υ is the scan rate, R is the gas constant, and T is the absolute temperature. The linear plots in Figure 2C are frequently taken as evidence that the electron transfer reaction is a diffusion-controlled process. It is evident from the data in Figure 2B that the peaks of the anodic and the cathodic currents appeared to be sliding apart as the scan rate increased. As the scan rate increased from 16 to 256 mV/s, the differences between the potentials associated with the respective, paired anodic and cathodic peaks increased from 150 to 308 mV, respectively. In addition, one would expect the reaction of the ferric/ferrous couple with the platinum electrode to be a one-electron exchange that would impose a limit of 59 mV on the difference between the voltages attendant with the anodic and cathodic peaks, a limit that is clearly exceeded in these data. These latter observations indicate that the electron transfer reaction is quasi-reversible in the electrochemical context.

It must be noted that chemical reversibility and electrochemical reversibility with an electrode have different physical meanings (Nicholson and Shain, 1964; Klingler and Kochi, 1981; Myland and Oldham, 2005). Chemical irreversibility means that the reactant or product is removed from future electron exchanges by an additional chemical reaction. Electrochemical irreversibility simply means that the electron transfer reaction with the electrode is very much slower than is the rate of diffusion of the soluble analyte to or from the electrode. If the rate of electron exchange with the electrode is rapid compared with the rate of diffusion of the soluble analyte, then the electrochemical reaction is said to be reversible. If the rate of reaction with the electrode and the rate of diffusion by the analyte are roughly comparable, then the electrochemical reaction is said to be quasi-reversible. The data in Figure 2B indicated that the electrochemical reaction was roughly comparable with the rate of diffusion of the soluble iron around the electrode; consequently, the conclusion was that the electron exchange between the soluble iron and the platinum electrode was quasi-reversible.

The total peak current plotted in Figure 2C is divided into component plots of the anodic (*upper line, circles*) and the cathodic (*lower line, triangles*) portions of the peak current in Figure 2D. Ferric and ferrous ions have slightly different aqueous diffusion coefficients of 7.19 and 6.04 × 10^–6^ cm^2^s^–1^, respectively (Gil et al., 1996). These and other appropriate values, including those taken from Figure 2D, were inserted into Equation 1 to obtain the value of 0.79 cm^2^ for the surface area of the platinum working electrode in these electrochemical measurements (detailed calculation shown in Supplementary Material). Even though it became evident that mobile, soluble iron was not required to mediate electron exchange between intact cells of *F. acidiphilum* and the solid working electrode (see below), these experiments with soluble iron did yield a value for the surface area of the working electrode that was required for subsequent calculations with the intact cells.

### Cyclic Voltammetry of Intact Cells

Cyclic voltammetry was conducted on washed suspensions of *F. acidiphilum* in sulfuric acid at pH 1.5. The *inset* of Figure 3A shows 5 cyclic voltammograms that were obtained at the same scan rate using 5 different suspensions of intact *F. acidiphilum*. The results were strikingly different from those obtained with soluble iron. Even though the number of cells in the suspensions varied over a 5-fold range from 0.48 to 2.4 × 10^9^ cells/ml in *curves a* to *e*, respectively, the resulting cyclic voltammograms were nearly indistinguishable. The prominent cathodic peak in each voltammogram was not accompanied by an equally prominent anodic peak. Instead, a putative anodic peak was barely distinguished as a slight swell in the baseline at around 1.0 V in the upper part of the traces. The cyclic voltammogram in the *inset* identified as *curve f* was obtained from the solution that remained when the intact cells were removed from the suspension by centrifugation, followed by filtration of the resulting supernatant through a filter with a 0.2 μM pore diameter. *Curve f* showed no indication of either an anodic or a cathodic peak. This observation suggested that the intact cells do not produce soluble, mobile extracellular electron transfer mediators in order to exchange electrons extracellularly with a solid electrode.

The *main panel* of Figure 3A shows the baseline-subtracted cathodic and anodic peaks that were extracted from their respective complete voltammograms. As anticipated from an inspection of the curves in the *inset*, the extents of both peak currents were nearly identical regardless of the number of intact cells in the suspension. Although reproducible cathodic peaks were extracted from the data in the *inset*, the currents that were associated with the anodic peaks were less than 10% of those of the corresponding cathodic peaks. These observations led to 2 hypotheses. First, the surface of the platinum working electrode is hypothesized to be rapidly coated with an adsorbed layer of intact cells, even when the lowest concentration of suspended cells was employed. The same cyclic voltammogram was obtained no matter whether the measurement was initiated within 4 or 40 min after introducing the cell suspension into the reaction cuvette with the 3 electrode system. There was simply insufficient time for any cellular adaptations to occur that might accompany the development of a mature biofilm on the surface of the platinum. The second hypothesis is that the weak anodic peak is a consequence of the competition for available electrons between the positive, oxidizing potential on the platinum electrode and the available molecular oxygen in the aerated suspension and present in the headspace of the cuvette that was open to the air. *F. acidiphilum* readily conducts aerobic respiration on soluble ferrous ions (Johnson et al., 2009), and it is plausible that the intact organism can actively respire using available electrons whether they are derived from soluble iron or from the cathodic wave imposed by a working electrode.

The *inset* of Figure 3B shows 3 representative cyclic voltammograms that were obtained using the same suspension of intact cells and 6 different sweep scan rates that varied over a 7-fold range. Each voltammogram had a strongly defined cathodic peak and a weakly defined anodic peak. The *main panel* of Figure 3B shows the baseline-subtracted anodic and cathodic peaks that were extracted from their respective voltammograms. The *bold arrow* passes through the peak values of the currents associated with each baseline-subtracted cathodic wave and indicates that the extents of the cathodic currents at their peaks increased as the scan rate increased.

A reduction potential for the intact *F. acidiphilum* was calculated by averaging the means of the potentials that corresponded with each set of anodic and cathodic peaks associated with each of the 11 voltammograms in Figures 3A,B (Supplementary Table 2). The average value for the reduction potential of *F. acidiphilum* in sulfuric acid at pH 1.5 was determined as 786 ± 14 mV vs. the SHE. It is fitting and proper that the apparent reduction potential of intact *F. acidiphilum* is more positive than that of the soluble iron that serves as the source of electrons when the organism is cultured by aerobic respiration on ferrous ions.

Figure 3C is a secondary plot of the total peak current as a function of the scan rate. The values on the *ordinate* represent the sums of the baseline-subtracted cathodic and anodic peaks that were extracted from the data in Figure 3B. The Randles-Sevcik equation does not apply here because the intact cells behave as though they rapidly adsorb to the surface of the platinum electrode. Instead, the linear relationship shown in Figure 3C is indicative of a surface adsorption process where the cells form a thin film on the surface of the electrode according to the following equation (Laviron, 1979):

(2) iP=nF2A2υΓ/RT

where Γ represents the quantity of adsorbed, electrochemically active sites per electrode surface area. The current varies as a direct function of the scan rate because the entire pool of accessible adsorbed cells can be completely oxidized or reduced during every unidirectional sweep of the potential through the working electrode. The additional observation from Figure 3A that further increases in the concentration of the cell suspension have no appreciable effect on the values of the maximum peak currents at any particular scan rate is consistent with the hypothesis that the cells constitute at least a monolayer-like coverage of the available surface area of the electrode.

Significant separation between the cathodic and anodic peak currents is evident from the data shown in Figures 3B,D shows a linear dependence of the difference in the potential between the corresponding cathodic and anodic peaks on the logarithm of the scan rate (Klingler and Kochi, 1981). The differences between the two potentials at their respective peaks varied from 500 mV when the scan rate was 36 mV/s to 740 mV when the scan rate was 256 mV/s. These peak potential separation values suggest that the exchange of electrons between the cells and the electrode is irreversible in the electrochemical context.

### Chronocoulometry of Soluble Iron

**FIGURE 4:**
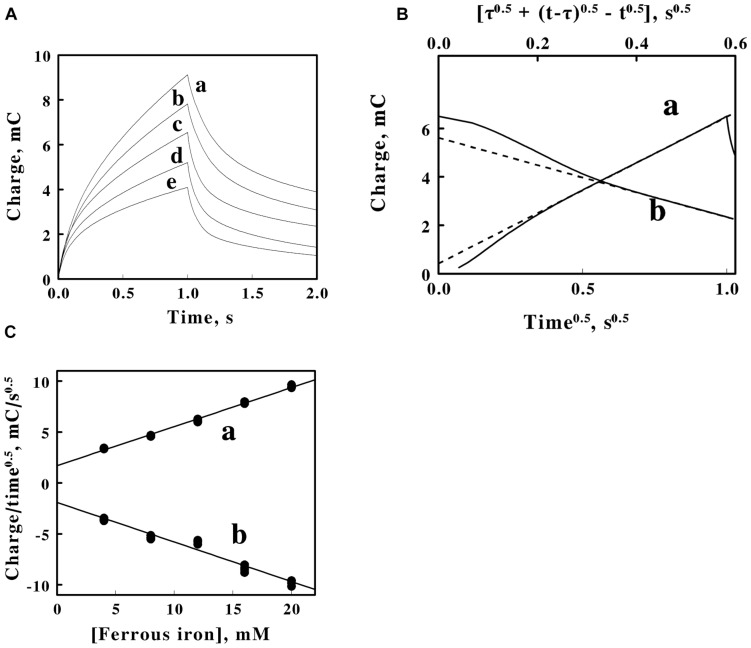
Double potential step chronocoulometry on soluble iron in sulfuric acid, pH 1.5. **(A)** Charge vs. time responses obtained with 5 concentrations of equimolar ferric and ferrous sulfate: 20, 16, 12, 8, and 4 mM soluble iron for *curves a–e*, respectively. Each experimental curve of the charge as a function of time represents the mean of five individual determinations whose values varied by less than 1% among the five measurements. The increase in charge is a reduction step; the subsequent decrease in charge is an oxidation step. **(B)** Representative Anson plots for the reduction (*curve a, lower abscissa*) and the oxidation (*curve b, upper abscissa*) potential steps obtained using 12 mM iron, showing both the charge values (*solid curves*) and the slopes (*dashed lines*) used to characterize the latter portions of the charge curves; t and τ represent the time since the start of the measurement and the duration of the first potential step, respectively. **(C)** Dependence of the slopes extracted from Anson plots, such as those shown in **B**, on the concentration of soluble iron for the reduction (*curve a*) and oxidation (*curve b*) potential steps in **A**. Each datum in **panel C** represents the mean of five determinations. The standard deviation of each datum in *curve a* was within the area bound by the diameter of the datum. The standard deviation of the data at each concentration of soluble iron in *curve b* varied from 3 to 3.7%.

Double potential step chronocoulometry measurements were conducted on both soluble iron and intact *F. acidiphilum* to augment and extend the cyclic voltammetry measurements summarized above. Chronocoulometry imposes a relatively large and immediate change, or step, on the potential applied to the working electrode (Anson, 1966; Anson and Osteryoung, 1983; Bott and Heineman, 2004). Figure 4A shows plots of the total charge exchanged as a function of time when different concentrations of soluble iron in sulfuric acid at pH 1.5 were subjected to two steps of different and opposite voltages. The first step in Figure 4A shows the charges that were transferred from the electrode to the soluble iron when the voltage that was imposed on the electrode was stepped for 1 s from 1.6 to 0.2 V s. the SHE. The second step represented in Figure 4A shows the charges that were transferred from the soluble iron back to the electrode when the voltage that was imposed on the electrode was stepped from 0.2 back to 1.6 V. The quantities of the charges increased as the concentration of soluble iron increased. The electrons transferred back to the electrode in the second step were always fewer than those deposited on the soluble iron in the first step due to diffusion of the recently reduced iron away from the immediate vicinity of the electrode.

Figure 4B shows representative Anson plots of the charge as a function of the square root of time for just one concentration of soluble iron. The Anson equation expresses the total charge as a function of the square root of time as follows (Anson and Osteryoung, 1983):

(3) Qf=Qdl+2nFACDt0.5/0.5π0.5

Where Q_*f*_ is the amount of charge in coulombs that has passed at time t since the initiation of the first potential step and Q_*dl*_ is the charge that was consumed by the electrode-electrolyte double layer capacitance when the voltage was changed from its initial to its final value. The Anson equation when applied to the second step takes the following form (Anson and Osteryoung, 1983):

(4) Q=rQ+dl2nFACD[τ+0.5(t-τ)-0.5t]0.50.5/π0.5

where τ is the time consumed by the first potential step, Q_*r*_ is the amount of charge that has passed since time τ, and t remains the overall time since the initiation on the first potential step. The *solid curves* in *a* and *b* of Figure 4B represent the primary data of the first, reductive step and the second, oxidative step, respectively. The respective *dashed lines* represent the corresponding tangents that were drawn to the latter portions of the primary data. The negative deviations at shorter times between the tangents and the actual charge values are primarily due to the inability of the potentiostat to instantaneously change the applied potential during the potential step.

Figure 4C shows the values of the tangents such as those illustrated by the examples in Figure 4B as a function of the total concentration of iron. A value of the charge needed to establish the double layer of ions in the solution adjacent to the surface of the electrode was extracted from the data in Figure 4C. The double layer charge taken from the intercepts of the two lines in Figure 4C was 1.8 ± 0.2 mC.

### Chronocoulometry of Cells

**FIGURE 5:**
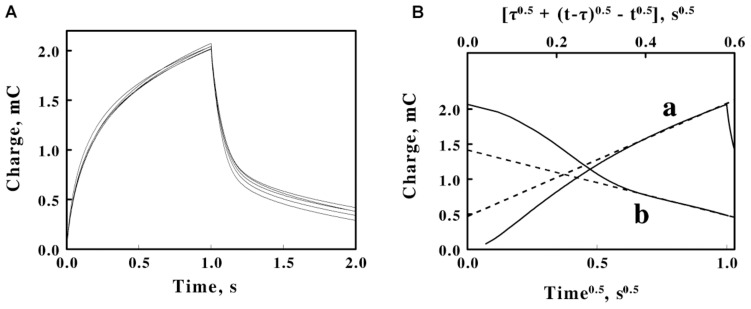
Double potential step chronocoulometry on suspensions of *F. acidiphilum* in sulfuric acid, pH 1.5. **(A)** Charge vs. time responses obtained with 5 suspensions of cells: 0.48, 0.96, 1.44, 1.92, and 2.4 × 10^9^ cells/ml. Each experimental curve of the charge as a function of time represents the mean of five individual determinations whose values varied by less than 1% among the five measurements. The increase in charge is a reduction step; the subsequent decrease in charge is an oxidation step. All 5 voltammograms are essentially superimposable. **(B)** Representative Anson plots for the reduction (*curve a, lower abscissa*) and the oxidation (*curve b, upper abscissa*) potential steps obtained using 1.44 × 10^9^ cells/ml, showing both the charge values (*solid curves*) and the slopes (*dashed lines*) used to characterize the latter portions of the charge curves; t and τ represent the time since the start of the measurement and the duration of the first potential step, respectively.

Double potential step chronocoulometry measurements were conducted on intact *F. acidiphilum* in sulfuric acid at pH 1.5. Figure 5A shows plots of the total charge exchanged as a function of time when different suspensions of washed *F. acidiphilum* were subjected to the same potential steps as those that were applied above to the soluble iron. As was observed in the cyclic voltammetry measurements featured in Figure 3A, varying the concentration of the cell suspensions over a 5-fold range had no appreciable effect on the experimental outcomes; the 5 experimental curves shown in Figure 5A are, for all practical purposes, identical. This observation is consistent with the hypothesis posed above based on similar observations shown in Figure 3A: that the platinum working electrode was rapidly coated with an adsorbed layer of intact cells, even when the lowest concentration of suspended cells was employed.

The data in each individual curve in Figure 5A conformed to the Anson equations. Figure 5B shows respective Anson plots of the charge as a function of the square root of time for just one suspension of *F. acidiphilum*. Supplementary Figure 2 shows the values of the tangents such as those illustrated by the examples in Figure 5B as a function of the concentration of cells in the reaction cuvette. Because the primary experimental curves in Figure 5A were nearly identical, the resulting nearly identical tangents derived from the Anson plots such as those in Figure 5B produced essentially horizontal lines in Supplementary Figure 2 that have no appreciable slopes. If the bulk of the electron exchange in these experiments is between the electrode and the adsorbed cells that cover the surface of the electrode, then electron exchange with cells that diffuse to the surface of the electrode would be insignificant, a hypothesis that is consistent with the horizontal lines in Supplementary Figure 2. The valuable observation from Supplementary Figure 2 is that the difference between the *ordinate intercepts* of the reductive (*a*) and oxidative (*b*) lines represents Q_*ads*_, the charge associated with reducing and oxidizing the cells that are immobilized in the adsorbed layer that covers the surface of the platinum electrode. The value of Q_*ads*_ is related to the surface concentration of the electrochemically active sites by the following relationship (Anson and Osteryoung, 1983; Bott and Heineman, 2004):

(5) Q=adsnFAΓ

Equation 5 permitted us to estimate the number of electrons passed per electrochemically active site on the working electrode. Using the values of Q_*ads*_ = 2.82 mC (Supplementary Figure 2), F = 1.6 × 10^–19^ C/electron, and A = 0.79 cm^2^ (from above), the value of nΓ, the multiplicand of the number of electrons passed per electrochemically active site multiplied by the electrochemically active sites per electrode surface area, was determined to be 3.7 × 10^–8^ moles or 2.23 × 10^16^ electrons-sites per cm^2^. Prior published studies used electrical impedance measurements to conclude that each cell of *F. acidiphilum* could be approximated as a sphere with an average diameter of slightly more than 0.5 μm (Blake et al., 2020). To a first approximation, one could pack 4 such cells onto each μm^2^ of a tightly packed monolayer of cells on the surface of the electrode. There are 10^4^ μm^2^ for every cm^2^. Consequently, each cm^2^ of electrode space could accommodate 4 × 10^8^ cells in such a tightly packed monolayer. If one permits 4 × 10^8^ cells/cm^2^ to represent the value of Γ in Equation 5, then n becomes 5.57 × 10^7^ electrons passed per electrochemically active site, which in this case represents a single adsorbed bacterium. This value is used below to estimate the average number of electrons that pass through each cytochrome in the cell during the cathodic phase of the cyclic voltammetry measurements.

### Spectroelectrochemistry of Cells

Supplementary Figure 3 shows a schematic diagram of the integrating cavity absorption meter that was employed to conduct spectrophotometric measurements on intact *F. acidiphilum* as it actively exchanged electrons with a platinum electrode. The observation cavity utilized for the absorbance measurements in Supplementary Figure 1 and for those *in situ* studies published elsewhere (Blake and Griff, 2012; Li et al., 2015; Blake et al., 2016, 2020; Blake and White, 2020) consisted of a hollow cavity that was filled with turbid suspensions of live cells. To conduct spectroelectrochemical measurements, we commissioned a customized observation chamber that could accommodate the standard-sized cuvette that doubled as an electrochemical reaction vessel in our measurements. The rectangular cavity in the new customized observation chamber was surrounded by a proprietary white particulate material that encouraged the random diffraction of the measuring light. To the extent that the resulting measuring light was purposefully scattered and highly diffuse, additional light scattering by turbid suspensions of live cells or the surfaces of 3 solid electrodes had no appreciable consequences on the integrity of the resulting absorbance measurements. The reaction cuvette with its 3 electrodes was simply inserted into the rectangular cavity of the customized observation chamber, and both electrochemical and absorbance measurements were conducted simultaneously on each sample.

**FIGURE 6:**
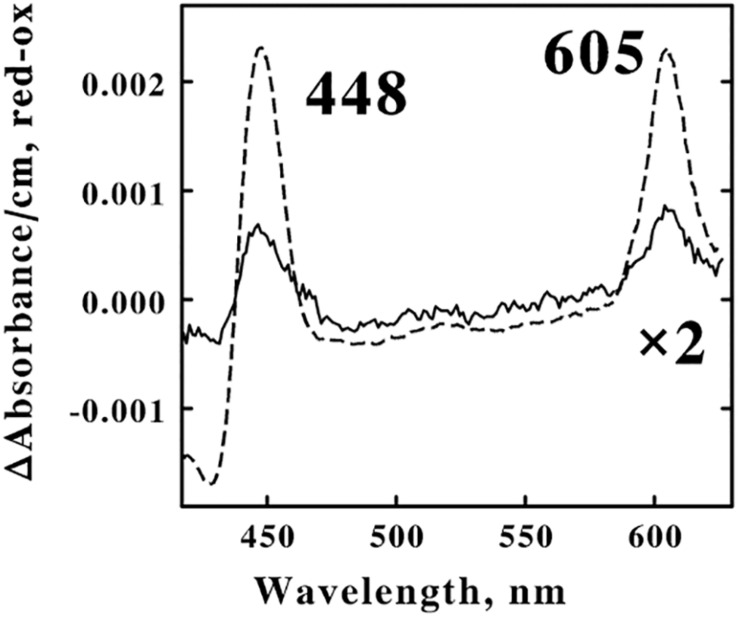
Identification of the principal electron transfer component that was reduced when intact *F. acidiphilum* was in the cathodic phase of repetitive cyclic voltammetry. Both *curves* are difference spectra representing the absolute spectrum of the reduced bacterium minus that of the air-oxidized bacterium. The *dashed spectrum* was obtained 0.5 s after 1.6 × 10^9^ cells/ml were mixed with 4.0 mM ferrous sulfate, pH 1.5 at 30 ^*o*^C. The *solid spectrum* was obtained from the cathodic portions (from 0.8 to 0.2 V vs. the SHE, inclusive) of multiple cyclic voltammetry measurements on 1.6 × 10^9^ cells/ml conducted in sulfuric acid, pH 1.5 at 30^*o*^C; the scan rate was 100 mV/s. ^1^ The abbreviations used are: SHE, standard hydrogen electrode; CV, cyclic voltammetry; CC, chronocoulometry; and tBLASTn, Basic Local Alignment Search Tool to search translated nucleotide databases using a protein query.

A suspension of *F. acidiphilum* that contained 1.6 × 10^9^ cells/ml was divided into 2 equal samples. The *dashed curve* in Figure 6 shows the difference spectrum of the absolute absorbance of the reduced bacterium minus that of the oxidized bacterium that was obtained when one sample was exposed to 4.0 mM ferrous sulfate in sulfuric acid, pH 1.5 at 30°C. The difference spectrum was characterized by reduced absorbance peaks at 448 and 605 nm, absorbance properties that are generally associated with terminal oxidases that catalyze the 4-electron reduction of molecular oxygen to 2 water molecules that constitutes the final reaction in aerobic respiratory chains. This reduced cytochrome 605 exhibited mathematical and correlational properties that were consistent with the hypothesis that oxidation of cytochrome 605 constituted the rate-limiting step when *F. acidiphilum* respired aerobically on soluble ferrous ions (Blake et al., 2020).

The *solid curve* in Figure 6 shows the corresponding difference spectrum that was obtained when the other sample was subjected to cyclic voltammetry in the absence of soluble iron in sulfuric acid, pH 1.5 at 30°C. After collecting a stable baseline in the absence of any applied potential, absorbance spectra were collected for 6 s during the cathodic wave as the applied voltage varied from 0.8 to 0.2 V vs. the SHE. The resulting difference spectrum represented 28% of the amplitude of the absorbance changes that were observed in the *dashed curve* when the identical suspension of cells was exposed to an excess concentration of ferrous ions. Twenty-eight percent of 1.6 × 10^9^ cells/ml is 4.5 × 10^8^ cells/ml. The microcuvette that was used in these electrochemical measurements accommodated only 0.8 ml of suspension; consequently a total of 0.8 × 4.5 × 10^8^ cells/ml, or just 3.6 × 10^8^ cells were electrochemically reduced in the cathodic phase represented in Figure 6. Recall that Equation 1 was utilized to estimate that each cm^2^ of the surface of the platinum electrode can accommodate 4 × 10^8^ cells in a tightly packed monolayer. Thus the present electrode with its surface area of 0.79 cm^2^ could therefore accommodate a maximum of 3.2 × 10^8^ cells. The agreement between the two estimates of the numbers of cells in the presumed monolayer, one estimated solely from the electrochemical measurements (3.2 × 10^8^) and one derived from the absorbance properties from the spectroelectrochemical measurements (3.6 × 10^8^), is entirely consistent with the hypothesis that only those cells that were in direct contact with the electrode actually received electrons directly from the solid platinum.

Finally, recall that the electrochemical measurements interpreted above yielded the number of electrons passed per electrochemically active site as 5.57 × 10^7^, where each “site” represents a single bacterium adsorbed onto the surface of the working electrode. Studies reported elsewhere found that each cell of *F. acidiphilum* harbors an average of 8.4 × 10^4^ cytochromes 605 (Blake et al., 2020). That means that there was an average of 660 electrons passed per cytochrome 605 in the reductive phase of the cyclic voltammetry. The data in Figures 3A, 5A were consistent with the hypothesis that the electrochemistry of *F. acidiphilum* actually supported an EC mechanism (Nicholson and Shain, 1964), where the reduction of the bacterium permitted the organism to respire aerobically using electrons derived from the electrode. If so, the data in Figures 3A,B indicate that about 90% of the electrons that were deposited in the adsorbed cells during the cathodic wave were not available to the electrode in the subsequent anodic wave. The turnover number for the aerobic oxidation of *in situ* cytochrome 605 was determined to be 35 s^–1^ when ferrous iron was the electron donor (Blake et al., 2020). If, for the sake of argument, one posits that oxidation of the cytochrome 605 occurred only during those 6 s as the electrode potential scanned from 0.8 to 0.2 V, then 90% of 6 s × 35/s equals 190 turnover events that can be attributed to each cytochrome 605 during that time if the cytochrome oxidase were operating at its maximum velocity. That level of maximum velocity in turnover would consume 760 electrons because the reduction of molecular oxygen to water requires 4 electrons per turnover event. That is more than sufficient capability to account for the 660 electrons passed per cytochrome 605 that were calculated above. Of course, the terminal oxidase would not necessarily be operating at its maximum velocity during the entire cathodic phase of the cyclic voltammetry measurement. The terminal oxidase would likely operate at a lower velocity both during the initial reduction phase until the cytochrome 605 population was fully reduced and after the applied potential switched directions and the reduced cytochrome 605 population decreased as the applied potential began to increase toward the anodic phase. Non-etheless, the electrochemical and spectroelectrochemical results are internally consistent with each other if the following hypotheses apply: (i) *F. acidiphilum* respires aerobically using electrons derived from the platinum electrode; and (ii) the oxidation of cytochrome 605 is also the rate-limiting step if *F. acidiphilum* respires aerobically when the platinum electrode serves as the electron donor.

## Discussion

*Ferroplasma acidiphilum* has three properties that make it an attractive candidate for spectroelectrochemical measurements using the intact organism. First, it is known to oxidize and dissolve extracellular, solid minerals like pyrite, FeS_2_ (Johnson et al., 2009). If it uses a direct mechanism to oxidize the solid pyrite (Tributsch, 2001; Crundwell, 2003; Zhang and Fang, 2005), then *F. acidiphilum* must necessarily express the biomolecules and/or infrastructure required to transport electrons from an extracellular source of electrons like pyrite across its outer cell wall to its plasma membrane, where aerobic respiration could proceed *via* oxidative phosphorylation per the Mitchell hypothesis. Secondly, it is known to both oxidize reduced soluble iron under aerobic conditions and reduce oxidized soluble iron under anaerobic or anoxic conditions (Johnson et al., 2009). If *F. acidiphilum* uses an indirect mechanism to oxidize pyrite (Tributsch, 2001; Crundwell, 2003; Zhang and Fang, 2005) where soluble, mobile iron simply shuttles electrons from the surface of the pyrite to the surface of the bacterium, then *F. acidiphilum* has a ready-made, physiological mediator to exchange electrons with any extracellular solid electrode that exchanges electrons with soluble ionic iron. Thirdly, *F. acidiphilum* is known to encode and express only two different terminal oxidases that contain *a*-type heme prosthetic groups (Blake and White, 2020; Blake et al., 2020). Consequently, any electrochemical-dependent spectral changes in the intact organism might be expected to be less complex than those that might be possible in a corresponding Gram-negative organism that expresses different colored biomolecules and prosthetic groups to transport extracellular electrons across an outer membrane and a periplasm to the plasma membrane (Li et al., 2015; Blake and White, 2020).

The capacity to exchange electrons with a solid working electrode has been reported for members of nearly 100 species (Koch and Harnisch, 2016, and references therein). The majority of existing reports on the interactions of microorganisms with solid electrodes have focused on the transfers of cellular electrons to an electropositive anode (Lovley, 2012, 2017a, b, and references therein). This emphasis is due, in part, to the decades-long availability of generous research funds from the U.S. Department of Energy to study the microbe-dependent reduction and immobilization of UO_2_^2+^ and other oxidized, mobile radionuclides within the earth’s anaerobic subsurface. These studies have focused primarily on the members of three Gram-negative genera: *Shewanella*, *Geobacter*, and *Desulfovibrio*. The environmental and physiological electron acceptors for these anaerobic and/or facultative bacteria are insoluble iron(III) oxides and soluble chelated ferric ions that are reasonably stable in the absence of molecular oxygen. The generally accepted mechanism(s) to conduct electrons from the interior of the cells to an extracellular electron acceptor like a solid electrode are thought to employ multiheme cytochromes *c* to conduct the intracellular electrons through the plasma membrane, across the periplasm in these Gram-negative organisms, and through the outer membrane to the outer surface of the cell (Patil et al., 2013; Garg et al., 2018). At that point, the electrons can be deposited directly onto the surface of an electropositive electrode from the outermost exposed cytochromes *c*. Alternatively, an extracellular mobile, soluble electron carrier can serve as an electron transfer mediator to shuttle electrons from the outermost cytochromes *c* to the solid electrode (Marsili et al., 2008; Edwards et al., 2015; Glasser et al., 2017). Although Gram-negative eubacteria that donate electrons to an anode have received the majority of the attention in laboratory settings, there are also reports that selected Gram-positive bacteria also conduct electron transfers to extracellular mobile electron transfer mediators and solid electrodes (Wrighton et al., 2008; Parameswaran et al., 2016). As an example, *Thermincola potens* JR is a thermophilic Gram-positive eubacterium that is hypothesized to exploit a nonaheme cytochrome *c* located at its outer cell surface as a terminal reductase to transfer electrons to a solid anode (Wrighton et al., 2011; Carlson et al., 2012; Costa et al., 2019).

The majority of the microorganisms that conduct anaerobic or anoxic respiration by donating electrons either directly or indirectly *via* an electron shuttle mediator to an electropositive anode have one thing in common: they all exploit insoluble iron oxides as electron acceptors in their natural, physiological environments. The standard reduction potentials of hematite (Fe_2_O_3_) and goethite (Fe-OOH) are −210 and −220 mV, respectively, vs. the SHE in 100 μM soluble iron at neutral pH (Gorski et al., 2016). The reduction potentials of these oxidized solids can vary over a ±100 mV range depending on the pH and the concentration of soluble iron. Examples of the mean standard reduction potentials of selected relevant multiheme cytochromes *c* that are expressed to transfer electrons from the organism to extracellular iron oxides are as follows: −220 and −212 mV for OmcZ and OmcS, respectively, expressed by *Geobacter sulfurreducens* (Inoue et al., 2010; Qian et al., 2011); −200 and −138 mV for CymA and MtrC, respectively, expressed by *Shewanella oneidensis* (Firer-Sherwood et al., 2008). Selected species of *Geobacter* and *Shewanella* have also been shown to synthesize and express extracellular flavins to serve the same purpose (Marsili et al., 2008; Edwards et al., 2015; Glasser et al., 2017). The standard reduction potentials of soluble FAD and riboflavin are −219 and −208 mV, respectively (Lowe and Clark, 1956; Harbury and Foley, 1958). The point here is that microorganisms that donate electrons to insoluble iron oxides possess the capability of expressing biopolymers and soluble electron transfer agents whose reduction potentials are the same or of similar magnitudes with those of the target iron oxide electron acceptors.

What about microorganisms that accept electrons from electronegative minerals or cathodes? If any microorganism that respires anoxically by donating electrons to an extracellular, insoluble oxide mineral is a good candidate to study electron transfer from the intact cell to an electropositive anode, then any microorganism that respires aerobically by accepting electrons from an extracellular, reduced mineral should be an equally good candidate to study electron transfer into an intact cell from an electronegative cathode. *F. acidiphilum* represents a group of microorganisms spread among 6 phyla that obtain their energy for growth from aerobic respiration on reduced sulfide minerals like pyrite under strongly acidic conditions (Blake et al., 2016; Blake and White, 2020). The standard reduction potential for pyrite is 620 mV in 1.0 M H_2_SO_4_, pH 1.0 (Peters and Majima, 1968; Chandra and Gerson, 2010). That value is approximately 800 mV more positive than are the standard reduction potentials for typical environmental iron oxides. A difference of 800 mV translates to a difference in affinity for a single electron of approximately 10^13^-fold, or 13 orders of magnitude. Consequently, the multiheme cytochromes *c* that are generally thought to donate electrons to extracellular iron oxides under anoxic conditions would, at first glance, appear to be poor choices for accepting electrons from extracellular pyrite, because their reduction potentials appear to be far too low to be compatible with that function. The complete genome of *F. acidiphilum* does indeed possess the information to express several types of *c*-type cytochromes (Blake and White, 2020), but none of these relevant open reading frames contain more than two of the canonical –CXXCH– cytochrome *c*-type heme-binding sequences per protein (unpublished observations). Further, tBLASTn searches (Altschul et al., 1997) that used the sequences of well-characterized multiheme cytochromes *c* from eubacteria that respire anoxically on iron oxides (Sharma et al., 2010) did not reveal any analogous sequences in the genome of *F. acidiphilum*. Consequently, *F. acidiphilum* does not appear to possess the capability to express multiheme cytochromes *c* to exchange electrons with its extracellular milieu. Rather, the results presented herein indicate that *F. acidiphilum* appears to interact with extracellular sources of high-reduction potential electrons using a transport chain that exhibits an empirical standard reduction potential of 786 mV.

Although it is evident that intact *F. acidiphilum* readily exchanges electrons with the surface of an extracellular platinum electrode, the means by which electrons are conducted through the presumably thick outer cell wall that surrounds this Gram-positive eubacterium are unknown. The only electrochemically active cellular protein that was visible during the cathodic phase of the cyclic voltammetry measurements was a cytochrome with reduced absorbance peaks at 448 and 605 nm. Previous studies identified this cytochrome as an *aa*_3_-type of heme copper terminal oxidase with a protein accession number of WP_052566320.1 (Blake et al., 2020). Because the sequence of this cytochrome is highly similar to the sequences of other *aa*_3_-type heme copper terminal oxidases in other acidophilic, iron-oxidizing chemolithotrophs (Blake and White, 2020), there is no reason to suspect that cytochrome 605 is not simply an integral membrane protein in the plasma membrane of *F. acidiphilum*.

So how does a cytochrome that is embedded in the plasma membrane conduct long-range electron exchanges with extracellular solids across a thick peptidoglycan wall? We propose that there are three possibilities. First, *F. acidiphilum* may express and secrete a series of biomolecules that contain electrically conductive prosthetic groups (not cytochromes *c*) that span the thick outer cell wall. Certain Gram-negative eubacteria express electrically conductive structures, dubbed “nanowires,” that were shown to be extensions of the outer membranes of Gram-negative bacteria that contain multiheme cytochromes that conduct electrons for relatively long distances to or from extracellular surfaces (Pirbadian et al., 2014). Structures with analogous functions known as conductive pili have been identified in both Gram-negative and Gram-positive microorganisms (Walker et al., 2018). However, tBLASTn searches of the *F. acidiphilum* genome were conducted using the sequences of canonical pilin proteins to no avail (unpublished observations). The second possibility is that *F. acidiphilum* secretes extracellular mobile, soluble electrochemically active molecules (Light et al., 2018) that mediate electron passage through the thick cell wall that is likely to be porous to solutes with molecular masses less than 1000 daltons (Scherrer and Gerhardt, 1973). However, no evidence for electrochemically active materials was obtained when cyclic voltammetry measurements were conducted herein using growth medium where intact cells were carefully removed (Figure 3A). The third possibility is that a network of soluble iron atoms is transiently retained in the interstitial spaces within the bacterium’s cell wall, and that these trapped iron atoms function as a conduit to conduct extracellular electrons through the cell wall. Bacterial cells that have been cultured for many passages in 20 mM or higher soluble iron retain readily quantified amounts of iron no matter how thoroughly they are washed in iron-free media (unpublished observations). There are large quantities of teichoic acids and teichoic and lipoteichoic phosphodiesters within the cell walls of Gram-positive bacteria (Brown et al., 2013**).** These acidic species possess pKa values from 2.9 to 3.5 (Cox et al., 1999). Even a protonated teichoic acid or a phosphodiester will just exhibit a lower affinity for cationic iron than does the corresponding negatively charged conjugate base. The premise is that electrons can freely hop among the network of soluble iron atoms that are bound and retained within the generally porous cell wall to function as a means to pass electrons through the cell wall. The concept of “electron hopping” among adjacent extracellular electron shuttles as an alternative to the actual diffusion of the electron shuttles has been hypothesized and discussed elsewhere (Glasser et al., 2017, and references therein). It is worth noting that the electrical potentials for the cathodic peaks observed herein with soluble iron and washed cells of *F. acidiphilum* were very similar, albeit not identical. Perhaps the electrochemical behavior of intact *F. acidiphilum* represents a weighted combination of trapped, retained iron atoms and cellular redox-active biopolymers.

## Data Availability Statement

The original contributions presented in the study are included in the article/Supplementary Material, further inquiries can be directed to the corresponding author/s.

## Author Contributions

RB wrote the manuscript, directed the project, and collected and interpreted the spectroelectrochemical measurements. ZW and AN directed and interpreted the electrochemical measurements. KS, NW, and BP cultured the bacteria and conducted the electrochemical measurements. All authors contributed to the article and approved the submitted version.

## Conflict of Interest

The authors declare that the research was conducted in the absence of any commercial or financial relationships that could be construed as a potential conflict of interest.

## Publisher’s Note

All claims expressed in this article are solely those of the authors and do not necessarily represent those of their affiliated organizations, or those of the publisher, the editors and the reviewers. Any product that may be evaluated in this article, or claim that may be made by its manufacturer, is not guaranteed or endorsed by the publisher.
