# Supplementary material for: Ferrimicrobium acidiphilum Exchanges Electrons With a Platinum Electrode via a Cytochrome With Reduced Absorbance Maxima at 448 and 605 nm
Source: Front Microbiol. 2021 Jul 26;12:705187. doi: 10.3389/fmicb.2021.705187 (PMC8350767; doi:10.3389/fmicb.2021.705187)
Supplement: Supplementary file 1 [file Data_Sheet_1.PDF]

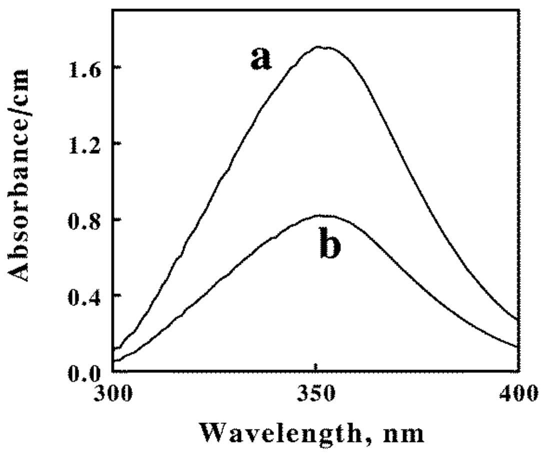

Figure S1. Oxidation and reduction of soluble iron by *Fm. acidiphilum*. *Spectrum a* was obtained after 2 mM ferrous sulfate in the appropriate growth medium was inoculated with *Fm. acidiphilum* and vigorously agitated for 72 hours at 30 ºC. *Spectrum b* was obtained after the same culture was incubated at 30 ºC for an additional 16 hours with no agitation.

Table S1. Standard reduction potential of soluble iron in sulfuric acid, pH 1.5, at 30 ºC. Data in the table are extracted from Figs. 3*A* and *B*. SHE is the standard hydrogen electrode.

Concentration of soluble iron, mM	Cyclic voltammetry scan rate, mV/s	Potential of anodic peak *vs*. SHE, mV	Potential of cathodic peak *vs*. SHE, mV	Mean of anodic and cathodic potentials at their peaks, mV
4	256	840	545	692.5
8	256	885	565	725
12	256	905	575	740
16	256	930	580	755
20	256	935	620	777.5
8	16	810	650	730
8	36	825	645	735
8	64	840	630	735
8	100	860	620	740
8	144	855	600	727.5
8	196	875	590	732.5
8	256	885	575	730

Average standard reduction potential for soluble iron = 735 ± 20 mV versus SHE.

Detailed calculation for the surface area of the platinum working electrode

The surface area of the platinum working electrode was calculated using the Randles-Sevcik equation (Equation 1 in the text) and data taken from Fig. 2*D*:

$$i_P = 0.4463 \text{ nFAC } (\text{nFD}\upsilon / \text{RT})^{0.5} \qquad (1)$$

The following identities were substituted into Equation 1: n = 1; F = 96,485.31 Coulomb/mol; R = 8.3145 V Coulomb/mol K; C = 8.0 mM; υ = 0.256 V/s; and T = 298.15 K. When the absolute value of the current difference at the respective cathodic peak in Fig. 2*D* (2.3341 mCoulombs/s) and the diffusion coefficient for ferric ions (7.19×10^{-6} cm^2/s) were also substituted into Equation 1, the value for the surface area of the platinum working electrode was determined to be 0.79 cm^2.

Table S2. Standard reduction potential of intact *Fm. acidiphilum* in sulfuric acid, pH 1.5, at 30 °C. Data in the table are extracted from Figs. 4*A* and *B*. SHE is the standard hydrogen electrode.

Concentration of cell suspension $\times 10^{-9}$ / ml	Cyclic voltammetry scan rate, mV/s	Potential of anodic peak *vs*. SHE, mV	Potential of cathodic peak *vs*. SHE, mV	Mean of anodic and cathodic potentials at their peaks, mV
0.48	256	955	610	783
0.96	256	925	605	765
1.44	256	935	605	770
1.92	256	925	610	767.5
2.40	256	945	605	775
1.44	36	920	660	790
1.44	64	930	665	798
1.44	100	960	645	803
1.44	144	970	630	800
1.44	196	975	625	800
1.44	256	980	610	795

Average standard reduction potential for intact *Fm. acidiphilum* = 786 ± 14 mV versus SHE.

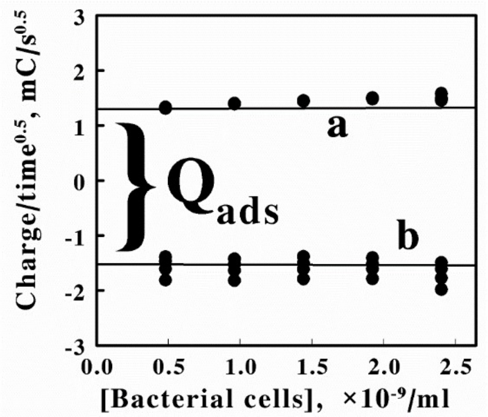

Figure S2. Dependence of the slopes extracted from Anson plots, such as those shown in Fig. 5*B*, on the concentration of cells for the reduction (*curve a*) and oxidation (*curve b*) potential steps in 5*A*. Q_{ads} represents the charge associated with reducing and oxidizing cells that are adsorbed onto the surface of the platinum electrode.

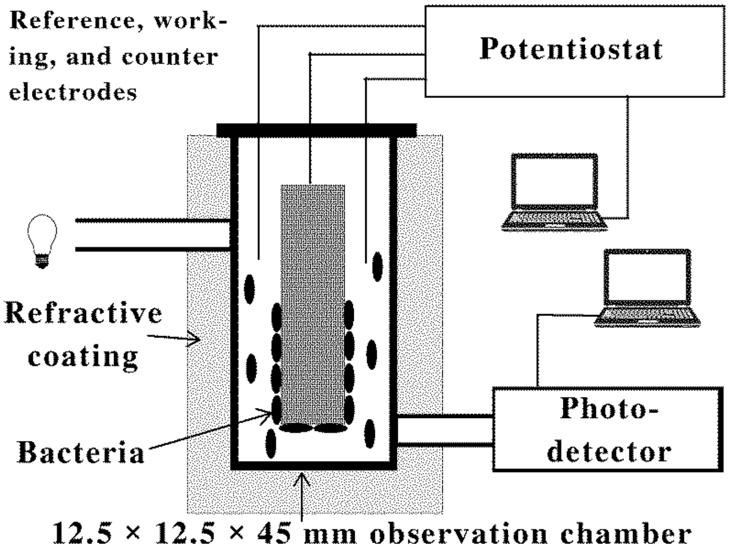

Figure S3. Schematic illustration of an integrating cavity absorption meter designed to conduct electrochemical measurements while simultaneously conducting accurate absorbance measurements in turbid suspensions of live microorganisms. The working electrode is a mesh comprised of platinum. The hollow observation chamber is built to accommodate a standard 3.5-ml quartz cuvette. Intact bacteria are depicted as both planktonic and adsorbed to the platinum working electrode.
